# Sedentary behavior patterns and bone health among overweight/obesity older women: a cross-sectional study

**DOI:** 10.3389/fmed.2024.1395790

**Published:** 2024-05-16

**Authors:** Yixian He, Litao Du, Qingqian Li, Xiaoyu Ren, Si Chen, Yang Pan

**Affiliations:** ^1^School of Physical Education, Shandong University, Jinan, China; ^2^Jinan Engineering Polytechnic, Jinan, China; ^3^School of Nursing and Rehabilitation, Cheeloo College of Medicine, Shandong University, Jinan, China

**Keywords:** sedentary behavior, bone, older women, overweight, obesity

## Abstract

**Background:**

Recent studies have indicated an association between sedentary behavior (SB), particularly patterns of SB, and bone health. However, it remains uncertain how different patterns of SB in overweight/obesity older women impact their bone health. This study aimed to investigate the association between objectively measured SB patterns and bone health in Chinese community-dwelling overweight/obesity older women.

**Methods:**

Cross-sectional data were obtained from a baseline survey of Physical Activity and Health in Older Women Study. Quantitative ultrasound was used to measure speed of sound (SOS), broadband ultrasound attenuation (BUA), bone quality index (BQI) and *T* value to evaluate bone health. SB patterns were measured using triaxial accelerometers, including sedentary time in SB bouts of ≥ 10, 30, and 60 min, number of SB bouts ≥ 10, 30, and 60 min. Multiple linear regression was used to examine the associations of different SB patterns with bone health.

**Results:**

After adjusting for confounders, sedentary time in SB bouts ≥ 60 min, number of SB bouts ≥ 60 min were significantly associated with bone health, with a lower SOS [β = −2.75, 95% confidence interval (CI): −4.96 to −0.53, *P* = 0.015], BUA (β = −1.20, 95% CI: −2.14 to −0.26, *P* = 0.013), BQI (β = −1.56, 95% CI: −2.63 to −0.49, *P* = 0.004), *T* value (β = −0.08, 95% CI: −0.14 to −0.03, *P* = 0.004) per 60 min increase of sedentary time in SB bouts ≥ 60 min, and a lower SOS (β = −3.97, 95% CI: −7.54 to −0.40, *P* = 0.029), BUA (β = −1.80, 95% CI: −3.44 to −0.16, *P* = 0.031), BQI (β = −2.28, 95% CI: −4.08 to −0.47, *P* = 0.014) and *T* value (β = −0.12, 95% CI: −0.22 to −0.03, *P* = 0.013) per bout increase of SB bouts ≥ 60 min, respectively.

**Conclusion:**

Limiting the duration of prolonged sedentary bouts and minimizing the occurrence of number of SB bouts ≥ 60 min could be essential in bone health management, especially for those older people who are overweight/obesity.

## 1 Introduction

Overweight/obesity is a risk factor for several chronic metabolic diseases and places a significant burden on the health management of older people. Bone health should not be ignored in this population. Overweight/obesity not only causes disk degeneration and increased prevalence of osteoarthritis by increasing mechanical loading (1, 2) but also exacerbates bone diseases such as osteoporosis and fractures by altering the internal cellular environment, resulting in metabolic abnormalities and increased inflammation (3, 4). Maintaining and improving bone health is a key factor in prolonging life for overweight/obesity seniors. It is clinically important to understand the association between behavioral factors and bone health in this population in order to prevent and rehabilitate bone disease at an early stage.

Sedentary lifestyle is a relatively modifiable risk factor that can be controlled by the older adults themselves (5). The World Health Organization has also advocated that older adults should limit sedentary behavior (SB) and increase physical activity (PA) to gain beneficial effects on population health and the prevention of osteoporosis (6). Several studies have found that SB is associated with worse bone health (7) and that total SB time is negatively correlated with bone mineral density (BMD) at different sites in women (8). In recent years, researchers have set out to further investigate the association between different patterns of SB and indicators of health outcomes, and have found that SB patterns are just as important for health (9). Scheers et al. (10) found that after adjustment for moderate-to-vigorous intensity PA (MVPA), the association between sedentary time in SB bouts ≥ 10 min and cardiometabolic risk was not significant (10). Hooker et al. (11) showed a 54% increased risk of stroke for sedentary time in SB bouts ≥ 17 min compared to sedentary time in SB bouts < 8 min. This is an indication that there are differences in the health effects of sedentary time in SB bouts. Thus, when discussing the association between SB and health, it is necessary to focus on the impact of SB patterns. It might be helpful to learn how different SB patterns are associated with bone health, which could help to change unhealthy lifestyles and reduce the risk of developing bone disease. However, there has been less research into the association of SB patterns with bone health, particularly in overweight/obesity older women. This study aims to investigate the association between objectively measured SB patterns and bone health in community-dwelling Chinese overweight/obesity older women. We hypothesize that longer and more frequent occurrences of prolonged sedentary bouts may have a greater and more negative impact on bone health.

## 2 Materials and methods

### 2.1 Participants

This study was based on STROBE cross-sectional reporting guidelines (12).

Cross-sectional data were obtained from the baseline survey of Physical Activity and Health in Older Women Study (PAHIOWS). A total of 1,370 participants were recruited in PAHIOWS, and the details can be found in our previous study (13). The present study was based on the overweight/obesity criteria for the Chinese population (14), overweight was defined as body mass index (BMI) ≥ 24 kg/m^2^ and < 28 kg/m^2^, and obesity was defined as BMI ≥ 28 kg/m^2^. Finally, 727 participants who were qualified with overweight/obesity criteria were included for evaluation. This study was approved by the Ethics Committee of the School of Nursing and Rehabilitation, Shandong University, China (2020-R-067), and all participants provided written informed consent.

### 2.2 Measurement of sedentary behavioral variables

Sedentary behavior patterns were evaluated by the triaxial accelerometer (ActiGraph wGT3X-BT, ActiGraph LLC, Pensacola, FL, USA) for 7 consecutive days. Raw data were processed using ActiLife software version 6.13.4. Non-wearing time was defined as ≥ 90 consecutive minutes of zero counts. The cut-off of SB was 0–99 counts per minute. Data from participants with at least 4 days of valid wearing time (at least 10 h of wearing time per day) were included in the analysis. Sedentary Behavior Research Network defined SB pattern as “The manner in which sedentary behavior is accumulated throughout the day or week while awake (e.g., the timing, duration and frequency of sedentary bouts, etc.)” (15). In this study, we investigated both total SB time (total accumulated SB time) and different sedentary bouts, including time accumulated in different sedentary bouts durations (the sum of time spent in accumulated bouts of SB lasting equal to or longer than 10, 30 or 60 min and total SB time) and frequency of different sedentary bouts durations occurs (the sum of frequency occur in accumulated bouts of SB lasting equal to or longer than 10, 30 or 60 min). All SB bouts were averaged per day. The confounding variables of total valid wearing time and MVPA (≥ 1,952 count per minute) as covariates were used to examine the independent effects of SB. Daily data were calculated for analysis.

### 2.3 Measurement of bone health

Quantitative ultrasound (QUS) was used in this study (16). Bone parameters were collected to characterize bone health by scanning the participants’ left heel bone with an ultrasound bone density analyzer, SONOST-2000. Participants were asked to take off their left footwear and the tester applied the coupling agent evenly to the participants’ left heel bone. The following bone parameters were measured: (1) speed of sound (SOS), which reflects flexibility and microstructure; (2) broadband ultrasound attenuation (BUA), which shows the correlation between the mineral content of the bone and BMD; (3) bone quality index (BQI), which is used to predict the risk of fracture (BQI = αSOS + βBUA, αβ: temperature corrections); and (4) *T* value, which is utilized to evaluate BMD and the extent of osteoporosis {*T* value = [BMD (subject)-Mean BMD (young adults)]/Standard Deviation of BMD (young adults)}.

### 2.4 Measurement of other variables

Considering the influence of other factors on the results (17–20), we collected data on potential confounders. Sociodemographic characteristics were collected by face-to-face interviews, including age, education level (primary and below, secondary, undergraduate and above), and drinking status (current drinker or not). Cognitive function was measured using the Chinese version of the mini-mental state examination (MMSE). Sleep quality was assessed using the Chinese version of the Athens Insomnia Scale (AIS). Nutritional status was measured using the mini nutrition assessment short form (MNA-SF). BMI and body fat rate (BFR) were measured using a body composition analyzer (MC-180, TANITA, Body Composition Analyzer, Japan). Hand grip strength (HGS) was measured using a hand dynamometer (Acmeway, Beijing, China), with two measurements taken and a minimum 15-s rest period between each, recording the maximum value from the two tests accurate to 0.01 kg.

### 2.5 Statistical analysis

Raw data were first processed using Excel software and then statistical analysis was performed using Stata 17 software. The normality of the variables was examined using the Kolmogorov-Smirnov test. Apply a logarithmic transformation to non-normally distributed variables to achieve normality. Continuous variables were expressed as mean ± standard deviation and categorical data were expressed as numbers and percentages. Multiple linear regression analysis was used to investigate the associations between total SB time, different patterns of SB (both accumulated time and number of different SB bouts) and bone parameters. Estimated β coefficients with 95% confidence interval (CI) were using linear regression in two models: model 1 was adjusted for age, education (triple categorical), body fat rate, hand grip strength, alcohol consumption (binary), insomnia (three categories), nutrition (binary), and accelerometer wearing time; model 2 was based on model 1 and additionally adjusted for total MVPA time. Variance inflation factors (VIF) were calculated for all variables to detect the presence of covariance. In the fully adjusted model 2, the VIF for each covariate was less than 3, which was considered acceptable. *P* < 0.05 was considered significant and *P* < 0.01 was considered highly significant.

## 3 Results

### 3.1 Baseline characteristics of the study population

The study included 727 participants with a mean age of 65 years. Of these, 67.8 percent were overweight and 32.2 percent were obesity, with mean BMI of about 27.2 kg/m^2^, and a mean body fat percentage of about 38.9. The total SB time per day was about 9.2 h, and sedentary time in SB bouts ≥ 10, 30, and 60 min were approximately 5.7, 2.9, and 1.3 h, respectively. The detailed characteristics of the study population are shown in Table 1.

**TABLE 1:** Characteristics of the total sample.

Characteristics	Total (*n* = 727)
Age (years)	65.0 ± 2.8
BMI (kg/m^2^)	27.2 ± 2.5
**Educational status [n (%)]**	
Primary school and below	100 (13.8%)
Middle school	508 (69.9%)
Junior college, undergraduate or above	119 (16.3%)
BFR (%)	38.9 ± 4.4
HGS (kg)	24.6 ± 5.2
**Drink status [n (%)]**	
No	662 (91.1%)
Yes	65 (8.9%)
**AIS [n (%)]**	
No sleep disorder	660 (90.8%)
Suspicious insomnia	43 (5.9%)
Insomnia	24 (3.3%)
**MNA [n (%)]**	
No	718 (98.8%)
Yes	9 (1.2%)
**Bone parameters**	
SOS (m/s)	1,494.4 ± 26.7
BUA (dB/MHz)	33.0 ± 12.5
BQI	63.9 ± 13.8
*T* value (g/cm^2^)	−2.2 ± 0.7
**Sedentary time (min/day)**	
In total	548.9 ± 121.9
In SB bout ≥ 10 min	343.5 ± 123.3
In SB bout ≥ 30 min	175.0 ± 99.9
In SB bout ≥ 60 min	77.6 ± 68.4
**Number of SB bouts (bouts/day)**	
SB bouts ≥ 10 min	13.8 ± 3.5
SB bouts ≥ 30 min	3.3 ± 1.6
SB bouts ≥ 60 min	0.9 ± 0.7
Total MVPA time (min/day)	30.5 ± 18.2
Accelerometer wearing time (min/day)	888.4 ± 121.5

Data shows mean ± standard deviation unless noted. AIS, Athens Insomnia Scale; BFR, body fat rate; BMI, body mass index; BQI, bone quality index; BUA, broadband ultrasound attenuation; HGS, hand grip strength; MNA, Mini Nutritional Assessment; MVPA, moderate-to-vigorous intensity physical activity; N, number; SB, sedentary behavior; SOS, speed of sound.

### 3.2 Associations between sedentary time in SB bouts and bone health

Table 2 displays the associations between sedentary time and bone parameters. Significant negative associations between sedentary time in bouts lasting ≥ 60 min and SOS, BUA, BQI and *T* value were found in all models (*P* < 0.05). Specifically, each additional 60 min per day of sedentary time in bouts lasting ≥ 60 min was associated with a 2.75 m/s lower SOS (β = −2.75, 95% CI: −4.96 to −0.53, *P* = 0.015), 1.2 dB/MHz lower BUA (β = −1.20, 95% CI: −2.14 to −0.26, *P* = 0.013), 1.56 lower BQI (β = −1.56, 95% CI: −2.63 to −0.49, *P* = 0.004) and 0.08 g/cm^2^ lower *T* value (β = −0.08, 95% CI: −0.14 to −0.03, *P* = 0.004). No significant associations were observed between total sedentary time, sedentary time in bouts lasting ≥ 10 min, ≥ 30 min and bone parameters after adjusting for potential confounders (*P* > 0.05).

**TABLE 2:** Associations between sedentary time and bone parameters.

Sedentary time (min/day)	Model	SOS β (95% CI)	*P*	BUA β (95% CI)	*P*	BQI β (95% CI)	*P*	*T* value β (95% CI)	*P*
Total	1	−1.15 (−2.79, 0.48)	0.166	−0.54 (−1.33, 0.25)	0.177	−0.67 (−1.53, 0.18)	0.122	−0.04 (−0.08, 0.01)	0.112
	2	−0.88 (−2.61, 0.84)	0.315	−0.33 (−1.41, 0.37)	0.252	−0.57 (−1.50, 0.36)	0.229	−0.03 (−0.08, 0.02)	0.217
In SB bouts ≥ 10 min	1	−1.03 (−2.31, 0.26)	0.118	−0.37 (−0.98, 0.23)	0.228	−0.54 (−1.20, 0.12)	0.108	−0.03 (−0.07, 0.01)	0.106
	2	−0.85 (−2.20, 0.50)	0.217	−0.34 (−0.99, 0.31)	0.303	−0.47 (−1.170, 0.23)	0.190	−0.03 (−0.06, 0.01)	0.191
In SB bouts ≥ 30 min	1	−1.42 (−2.93, 0.08)	0.064	−0.32 (−0.99, 0.36)	0.361	−0.62 (−1.37, 0.13)	0.104	−0.03 (−0.07, 0.01)	0.100
	2	−1.25 (−2.83, 0.33)	0.120	−0.27 (−0.98, 0.44)	0.460	−0.54 (−1.32, 0.25)	0.181	−0.03 (−0.07, 0.01)	0.178
In SB bouts ≥ 60 min	1	−2.92 (−5.05, −0.78)**	0.008	−1.22 (−2.13, −0.31)**	0.008	−1.63 (−2.66, −0.60)**	0.002	−0.09 (−0.14, −0.03)**	0.002
	2	−2.75 (−4.96, −0.53)*	0.015	−1.20 (−2.14, −0.26)*	0.013	−1.56 (−2.63, −0.49)**	0.004	−0.08 (−0.14, −0.03)**	0.004

CI, confidence intervals; SB, sedentary behavior. Model 1 adjusted for accelerometer wearing time, age, alcohol consumption (binary), body fat rate, education (triple categorical), grip strength, insomnia (three categories) and nutrition (binary). Model 2 additionally adjusted for Model 1 variables plus total moderate to vigorous physical activity time. *P < 0.05; **P < 0.01.

### 3.3 Associations between number of SB bouts and bone health

Table 3 displays the associations between number of different sedentary bouts and bone parameters. Significant negative associations between the number of SB bouts ≥ 60 min and SOS, BUA, BQI and *T* value were found in all models (*P* < 0.05), while there were no significant associations between the number of SB bouts ≥ 10 and 30 min and bone parameters (*P* > 0.05). Every additional increase of SB bouts ≥ 60 min was associated with a 3.97 m/s lower SOS (β = −3.97, 95% CI: −7.54 to −0.40, *P* = 0.029), 1.8 dB/MHz lower BUA (β = −1.80, 95% CI: −3.44 to −0.16, *P* = 0.031), 2.28 lower BQI (β = −2.28, 95% CI: −4.08 to −0.47, *P* = 0.014), and 0.12 g/cm^2^ lower *T* value (β = −0.12, 95% CI: −0.22 to −0.03, *P* = 0.013). These findings suggested that prolonged uninterrupted SB may have detrimental effects on bone health.

**TABLE 3:** Associations between number of sedentary bouts and bone parameters.

Number of sedentary bouts (bouts/day)	Model	SOS β (95% CI)	*P*	BUA β (95% CI)	*P*	BQI β (95% CI)	*P*	*T* value β (95% CI)	*P*
SB bouts ≥ 10 min	1	−0.20 (−0.90, 0.50)	0.567	−0.17 (−0.52, 0.18)	0.339	−0.17 (−0.55, 0.21)	0.375	−0.01 (−0.03, 0.01)	0.365
	2	−0.09 (−0.80, 0.62)	0.808	−0.15 (−0.52, 0.22)	0.422	−0.12 (−0.51, 0.27)	0.538	−0.01 (−0.03, 0.01)	0.529
SB bouts ≥ 30 min	1	−0.79 (−2.32, 0.74)	0.311	0.07 (−0.64, 0.79)	0.843	−0.19 (−0.98, 0.59)	0.630	−0.01 (−0.05, 0.03)	0.613
	2	−0.57 (−2.16, 1.02)	0.480	0.15 (−0.60, 0.90)	0.696	−0.08 (−0.89, 0.74)	0.855	−0.01 (−0.05, 0.04)	0.841
SB bouts ≥ 60 min	1	−4.27 (−7.73, −0.82)*	0.015	−1.85 (−3.43, −0.27)*	0.022	−2.40 (−4.15, −0.65)**	0.007	−0.13 (−0.22, −0.04)**	0.007
	2	−3.97 (−7.54, −0.40)*	0.029	−1.80 (−3.44, −0.16)*	0.031	−2.28 (−4.08, −0.47)*	0.014	−0.12 (−0.22, −0.03)*	0.013

CI, confidence intervals; SB, sedentary behavior. Model 1 adjusted for accelerometer wearing time, age, alcohol consumption (binary), body fat rate, education (triple categorical), grip strength, insomnia (three categories) and nutrition (binary). Model 2 additionally adjusted for Model 1 variables plus total moderate to vigorous physical activity time. *P < 0.05; **P < 0.01.

## 4 Discussion

To the best of our knowledge, our study was the first to explore the association between SB patterns and bone health in overweight/obesity older women. The results showed that only sedentary time in SB bouts ≥ 60 min and number of SB bouts ≥ 60 min were significantly and negatively associated with bone health. However, sedentary time in SB bouts ≥ 10 and 30 min and number of SB bouts ≥ 10 and 30 min were not significantly associated with bone health in this population. Therefore, our study suggests that limiting prolonged SB bout duration in daily life may prevent bone disease from developing in overweight/obesity older women.

Sedentary behavior is a common behavior in modern daily life which is a risk factor for bone health (21). In this study, we found that the total SB time per day in overweight/obesity older women was about 9.2 h. This finding is consistent with that of Shiroma et al. (22) who measured 3,565 older women aged 60–70 years and found that their total SB time per day was 9.5 h. In addition, Gennuso et al. (23) measured 1,914 older adults aged ≥ 65 years and found total SB time of 9.4 h per day, which is closer to the results of this study (23). Our regression results showed that the total SB time was not associated with any of the bone parameters. Previous research has found no association between total SB time and BMD at the lumbar spine and hip (24), which is consistent with the present study. However, total SB time has also been found to be associated with decreased femoral neck bone mass in postmenopausal women (25). As well, LaMonte et al. (26) found a positive association between total SB time and fracture risk in postmenopausal women, which is not consistent with our study (26). The reasons for differences in results from other previous studies are multifaceted which include heterogeneity in the population, sample size, and adjustment for covariates, as well as the measurement of bone health. Furthermore, although this study found no association with total SB time, it did find an association with SB patterns. Consequently, it is possible that SB patterns may have a more significant impact on bone health in overweight/obesity older women.

Although several studies have investigated the association between SB patterns and health outcome indicators (11, 27–29), there is less evidence to support the discussion of bone health, particularly among overweight/obesity older women. Our study found an independent effect of SB on bone health of overweight/obesity older women, specifically for sedentary time in SB bouts ≥ 60 min. For SB bout duration, Chastin et al. (8) who analyzed National Health and Nutrition Examination Survey (NHANES) data in the United States reported that mean SB bout duration was negatively associated with total femur and hip BMD in women, whereas our study further categorized SB bout duration into sedentary time in SB bouts ≥ 10, 30, and 60 min and showed that only sedentary time in SB bouts ≥ 60 min was significantly negatively associated with bone health. This is consistent with the findings of Gobbo et al. (30), who measured proximal femur and lumbar spine BMD using dual energy X-ray absorptiometry (DEXA) in 68 Brazilian older adults over 60 years of age with two years of SB follow-up, and found that limiting prolonged SB, especially for lasting ≥ 1 h, may have a beneficial effect on BMD. In addition, a study by Onambele-Pearson et al. (31) collected data on participants’ SB for sedentary time in SB bouts < 5 min, > 5 min, and measured BMD of the ribs, spine, pelvis, upper and lower extremities, and whole body using DEXA, which showed a significant positive correlation between sedentary time in SB bouts < 5 min and BMD of the ribs and lower extremities, suggesting that interrupting a continuous SB bout duration has a positive effect on BMD, which also corroborates our results.

For number of SB bouts, we found an association between number of SB bouts ≥ 60 min and bone health, such that each additional number of SB bouts ≥ 60 min led to worse bone health. Despite the paucity of available evidence, one study (25) found an independent association between the number of SBs and osteoporosis by analyzing bone health at different sites in 44 postmenopausal women. Another study encourages older women with bone disease to reduce number of SB bouts, with SB bout duration lasting ≥ 20 min, to protect bone health (30). Overall, our results suggest that reducing number of SB bouts ≥ 60 min is beneficial for bone health of overweight/obesity older women.

It is physiologically plausible that SB contributes to bone mass loss in overweight/obesity populations, and the potential mechanisms are complex. It has been shown that the bone cells of the organism have a pressure-sensing function and that they can be greatly affected by mechanical stress (32). Then, when SB occurs in overweight/obesity older adults, their lower extremities remain flaccid and weightless for long periods, so bone cells are unable to sense whole-body weight changes, leading to loss of bone mass in the lower extremities. However, during activities such as running, standing, and jumping, the bone cells of the lower extremities can feel the full weight of the body, and the bones are subjected to continuous weight-bearing stimulation, and PA can reduce the levels of pro-inflammatory factors (TNF-α and IL-6) produced by large amounts of adipose tissue in the body of obese people, and reduce their promoting effects on osteoclasts (33), thus inhibiting bone loss and contributing to the safeguarding of bone health levels. In conclusion, although it is not possible to infer reverse causality in this study, we can still support the idea that SB may be a risk factor for bone health. Therefore, the overweight/obesity older population should be encouraged to limit prolonged SB bout duration and reduce number of SB bouts ≥ 60 min, thereby increasing the effects of mechanical loading on bone cells (34, 35) and preventing the development of bone disease. As well, these findings will be of benefit to caregivers in scientifically and effectively guiding older adults who have decreased understanding and learning in the delivery of targeted bone healthcare.

This study has several limitations. First, this study was based on a cross-sectional design, which could not confirm causality, more randomized controlled trials and longitudinal studies are needed to further demonstrate the association between SB patterns and bone health in overweight/obesity older population. Secondly, QUS has certain limitations in assessing BMD. Thirdly, the participants included in this study were only individuals from one city in China, thus it is not representative of the entire Chinese overweight/obesity older population. Fourthly, the participants included were all volunteering and therefore likely to be relatively healthier. Finally, the accelerometer may be biased in the assessment of PA because it (1) excludes any activity in water since it is not waterproof and (2) is not as reliable in acquiring cycling movements.

## 5 Conclusion

Our study suggests that there may be a negative association between prolonged SB bouts lasting ≥ 60 min and bone health in overweight/obese older women. This finding highlights the importance of limiting continuous SB and reducing the number of SB bouts as a potential strategy for maintaining or improving bone health. However, since our study is a cross-sectional design, it cannot prove a causal relationship between SB patterns and bone health. Therefore, we recommend conducting more prospective studies and randomized controlled trials to investigate the causal association and determine the hazardous volume for prolonged SB bout duration.

## Data availability statement

The raw data supporting the conclusions of this article will be made available by the authors, without undue reservation.

## Ethics statement

The studies involving humans were approved by the Ethics Committee of the School of Nursing and Rehabilitation, Shandong University, China. The studies were conducted in accordance with the local legislation and institutional requirements. The participants provided their written informed consent to participate in this study.

## Author contributions

YH: Conceptualization, Investigation, Writing – original draft, Writing – review & editing. LD: Formal analysis, Writing – original draft, Writing – review & editing. QL: Data curation, Supervision, Writing – review & editing. XR: Data curation, Formal analysis, Writing – review & editing. SC: Methodology, Writing – review & editing. YP: Conceptualization, Supervision, Writing – review & editing.
